# L-arginine supplementation reduces mortality and improves disease outcome in mice infected with *Trypanosoma cruzi*

**DOI:** 10.1371/journal.pntd.0006179

**Published:** 2018-01-16

**Authors:** Sofía Carbajosa, Héctor O. Rodríguez-Angulo, Susana Gea, Carlos Chillón-Marinas, Cristina Poveda, María C. Maza, Diana Colombet, Manuel Fresno, Núria Gironès

**Affiliations:** 1 Centro de Biología Molecular Severo Ochoa, Consejo Superior de Investigaciones Científicas (CSIC), Universidad Autónoma de Madrid (UAM), Cantoblanco, Madrid, Spain; 2 Instituto Venezolano de Investigaciones Científicas (IVIC), Caracas, Venezuela; 3 Centro de Investigaciones en Bioquímica e Inmunología (CIBICI-CONICET). Departamento de Bioquímica Clínica. Facultad de Ciencias Químicas, Universidad Nacional de Córdoba, Córdoba, Argentina; 4 Instituto Sanitario de Investigación Princesa. Madrid, Spain; Instituto de Investigaciones Biotecnológicas, ARGENTINA

## Abstract

Chagas disease caused by *Trypanosoma cruzi* is a neglected disease that affects about 7 million people in Latin America, recently emerging on other continents due to migration. As infection in mice is characterized by depletion of plasma L-arginine, the effect on infection outcome was tested in mice with or without L-arginine supplementation and treatment with 1400W, a specific inhibitor of inducible nitric oxide synthase (iNOS). We found that levels of L-arginine and citrulline were reduced in the heart and plasma of infected mice, whereas levels of asymmetric dimethylarginine, an endogenous iNOS inhibitor, were higher. Moreover, L-arginine supplementation decreased parasitemia and heart parasite burden, improving clinical score and survival. Nitric oxide production in heart tissue and plasma was increased by L-arginine supplementation, while pharmacological inhibition of iNOS yielded an increase in parasitemia and worse clinical score. Interestingly, electrocardiograms improved in mice supplemented with L-arginine, suggesting that it modulates infection and heart function and is thus a potential biomarker of pathology. More importantly, L-arginine may be useful for treating *T*. *cruzi* infection, either alone or in combination with other antiparasitic drugs.

## Author summary

*Trypanosoma cruzi* is the causative agent of the neglected Chagas disease in humans. During infection in mice, depletion of plasma L-arginine is correlated with mortality. L-arginine is a semi-essential amino acid needed for cell proliferation, and is the substrate of arginase 1 (Arg-1) and inducible nitric oxide synthase (iNOS), which is involved in the immune response against infections. Observed L-arginine depletion is likely caused by increased Arg-1 activity, but the effect on immune response are still unknown. Our hypothesis is that L-arginine depletion may block nitric oxide (NO) production by iNOS, which is needed for parasite killing. To test this hypothesis, mice were supplemented with and without L-arginine, and the differential effect of treatment with an iNOS inhibitor was determined. L-arginine supplement was beneficial to the mice, lowering mortality and improving disease outcome and heart function. The beneficial effect was associated with increased levels of NO, thus low levels of L-arginine and NO are considered candidate markers of pathology. Finally, as L-arginine is a common dietary supplement, it may be useful for treatment of Chagas patients, either alone or in combination with antiparasitic drugs.

## Introduction

Chagas disease (or American Trypanosomiasis) is caused by *Trypanosoma cruzi*, a protozoan parasite of the *Kinetoplastidae* family [1]. About 7 million people are affected, with 100 million at risk of infection in 21 Latin American countries. Currently, Chagas disease is considered by the WHO as a neglected tropical disease [2], though several cases of Chagas disease have been reported outside of Latin America, in countries such as Spain [3] and the USA [4] due to migration. Treatment with antiparasitic drugs is effective during the acute phase, but not in the chronic phase, where it presents many undesirable secondary effects. Chagasic cardiomyopathy is the most common cause of disability in chronically infected patients, and unfortunately, treatment with benznidazole in chronic patients has shown low effectiveness [5]; thus, there is a need to progress towards new therapies and biomarkers of pathology.

We have previously described that during *T*. *cruzi* infection, there is infiltration by monocytic myeloid-derived suppressor cells (M-MDSCs) in cardiac tissue. M-MDSCs are characterized by their expression of arginase 1 (Arg-1) and inducible nitric oxide synthase (iNOS) and their ability to suppress T cell proliferation. Remarkably, high levels of Arg-1 expression have been correlated with L-arginine depletion [6], in agreement with other reports that arginase activity is the main cause of the low availability of L-arginine for nitric oxide (NO) production by iNOS [7]. Thus, administration of dietary L-arginine may be beneficial for the host during *T*. *cruzi* infection.

L-arginine is considered semi-essential in mammals, as dietary supplementation is needed during stressful conditions such as pregnancy, trauma or infection, during which the requirements exceed the production capacity of the organism [8]. L-arginine metabolism is a complex biological process, as it serves as the substrate of several enzymes. Arg-1 catalyzes conversion of L-arginine to L-ornithine which subsequently converts into L-proline, responsible for collagen and polyamine synthesis necessary for cell proliferation. L-arginine is also metabolized by iNOS for the production of citrulline and NO, [9, 10] which is capable of killing the parasite [11]. In fact, L-arginine enhanced the NO-dependent killing of intracellular *T*. *cruzi* in murine peritoneal macrophages [12].

Regulation of iNOS activity is a similarly complex process. Asymmetric dimethylarginine (ADMA) is generated by catabolism of proteins with methylated arginine residues [13], and is an endogenous inhibitor of iNOS [14]. Low levels of L-arginine causes down-regulation of iNOS expression [15] and reduced NO production by substrate competition [16]. Thus, there is cross-regulation of enzymatic activity by the different products of L-arginine metabolism [17].

We previously described that L-arginine supplementation during *T*. *cruzi* infection decreases parasite burden in mice [6]. Here we analyzed L-arginine-related metabolites from infected mice, observing increased ADMA and a reduction in citrulline and arginine levels in plasma and heart tissue, which reflects reduced iNOS activity, pointing to all of them as potential biomarkers of pathology. More importantly, L-arginine supplementation was found to increase survival and improve cardiac performance (assessed by electrocardiography) in infected mice, suggesting that it may be useful as a treatment, either alone or in combination with antiparasitic drugs.

## Methods

### Parasites and mice

BALB/c mice (6–8 week-old) were purchased from Harlan-Interfauna Iberica and Charles River Laboratories España, and maintained at the animal facility of the Centro de Biología Molecular Severo Ochoa (CBMSO, CSIC-UAM, Madrid, Spain) animal facility. For some experiments, mice were maintained at the Animal Resource Facility of the Centro de Investigaciones en Bioquímica e Inmunología (CIBICI-CONICET, Córdoba, Argentina) and at the Instituto Venezolano de Investigaciones Científicas (IVIC, Caracas, Venezuela). *In vivo* infections were performed with strain Y of *T*. *cruzi* as described previously [6, 18]. Groups of 6 mice were infected by intraperitoneal (IP) injection with 2,000 blood trypomastigotes per mouse, except when otherwise indicated. Evolution of such an infection is characterized by high parasitemia (usually with two peaks observed during the second and third weeks post-infection), with 100% mortality in BALB/c mice by 30 days post-infection (d.p.i.) when 2,000 parasites are inoculated per mouse. With a lower inoculum (50 parasites/mouse), parasitemia is lower and the survival rate is around 60%. Groups of 3–6 non-infected control mice were included in each experiment. Survival was monitored daily and parasitemia levels were checked every 2–3 days by the Brener method [19]. Clinical disease scores were determined by visual evaluation of parameters such as stooped posture, bristly back hair, presence of ventral urine stains and lack of activity, assigning a numerical value from 0 (minimal symptoms) to 4 (maximum symptoms). Blood samples were collected periodically and tissue samples were collected after animals were euthanized at the end of the experimental period. When indicated, drinking water was supplemented with fresh L-arginine mono-hydrochloride (Sigma-Aldrich) every other day to a final concentration of 3.75 mg/ml, and 20 mg/kg of the iNOS-specific inhibitor 1400W (Sigma-Aldrich) [20] was administrated daily by IP injection.

### Ethics statement

This study was carried out in strict accordance with the European Commission legislation for the protection of animals used for scientific purposes (directives 86/609/EEC and 2010/63/EU). Mice were maintained under specific pathogen-free conditions at the CBMSO (CSIC-UAM) animal facility. The protocol for the treatment of the animals was approved by the “Comité de Ética de Investigación” of the Universidad Autónoma of Madrid, Spain (permits CEI-14-283 and CEI-47-899). Experiments performed in Argentina followed the recommendations in “The Guide for the Care and Use of Experimental Animals” (Canadian Council on Animal Care). Animal handling and experimental procedures were approved by the Institutional Experimentation Animal Committee of The National University of Córdoba (permit 388/11), and animals were maintained at the Animal Resource Facility of the CIBICI-CONICET (NIH-USA assurance number A5802–01). Experiments performed in Venezuela were in strict accordance with “Bioethics and Biosafety Norms” (3rd edition) approved by Fondo Nacional de Ciencia y Tecnología de Venezuela (FONACIT), Ministerio de Ciencia y Tecnología of Venezuela (2011), the Asociación Venezolana para la Ciencia de los Animales de Laboratorio, and “International Ethical Standards for Research Biomedical in Animals of the WHO” (1982); animal handling and experimental procedures were approved by the Comité de Bioética Institucional (permit DIR-0031/1582/2017). Animals had unlimited access to food and water, and at the conclusion of the studies were euthanized in a CO_2_ chamber with every effort made to minimize their suffering.

### Analysis of metabolites

Concentration of nitrites (NO_2_Na), indicative of NO production, was measured in plasma and cell culture supernatants using the Griess reagent following the directions of the manufacturer (Sigma-Aldrich). When indicated, ornithine, urea, proline, putrescine, citrulline, ADMA and L-arginine levels were determined in mouse tissue extracts by Metabolon Inc., and expressed as ScaledImpData as previously described [21]. L-arginine level in plasma was determined after centrifugation at 20,800 g to remove protein precipitates, and 5 μl was analyzed using an HPLC chromatograph coupled to a triple quadrupole mass spectrometer (Varian 1200L; Agilent Technologies) as previously described [6]. ADMA concentration was determined using the mouse ADMA ELISA kit following the directions of the manufacturer (Cusabio).

### Protein expression analysis

Protein extracts were prepared from heart tissue perfused with PBS containing 1 IU/ml of heparin, cut into small pieces using a sterile scalpel blade followed by mechanical disruption using a PT1300D homogenizer (Kinematica Polytron, Fisher Scientific) in Triton X-100-based protein lysis buffer as previously described [18]. For western blot, 15 or 50 μg of tissue extract was fractionated by SDS polyacrylamide gel electrophoresis and transferred to a nitrocellulose membrane (Hybond-ECL, Amersham Biosciences), stained with Ponceau (Pierce), photographed and blocked in 5% fat-free milk or 5% BSA in 0.1% Tween-20 Tris-buffered saline. Membranes were incubated overnight at 4–8°C with a 1:1,000 dilution of rabbit anti-iNOS (sc-50) or rabbit anti-Arg-1 (sc-18354) from Santa Cruz Biotechnology. Then, membranes were incubated with horseradish peroxidase-conjugated anti-rabbit IgG (Thermo Scientific), and detection was carried out with Supersignal detection reagent (Pierce) followed by photographic film exposure.

### Macrophage infection

Pelleted RAW 264.7 macrophages were resuspended in complete RPMI 1640 with 5% FBS, with or without 100 μM L-arginine supplement, and infection was done with 10 trypomastigotes per macrophage (ratio 10:1). After 72 h post-infection (h.p.i.), nitrite levels were determined in culture supernatants as described above, and parasites were quantified by microscopic observation of culture supernatants in a Neubauer chamber at 7 d.p.i.

### Histological studies

As previously described [22], hearts were collected from mice at 14 d.p.i. and placed in 10% neutral buffered formalin for at least 4 h at room temperature, followed by incubation in 70% ethanol overnight. Samples were then embedded in paraffin (Tissue Embedding Station Leica EG1160), and 5 μm tissue sections were prepared (Microtome Leica RM2155), dewaxed and rehydrated, stained with H&E staining and mounted permanently in Eukitt´s quick-hardening mounting medium (Biochemika, Fluka analytical). Sections were analyzed on a Leica microscope using the 20-40x magnification objectives.

Alternatively, hearts from mice were collected and fixed in 4% paraformaldehyde in PBS for 2 h at room temperature, followed by incubation in a 30% sucrose solution at 4°C overnight as described [18]. Tissues were then embedded in Tissue-Tek OCT in Cryomolds (Sakura), frozen in dry ice, stored at -80°C, and 10 μm sections were cut using a cryostat Leica CM1900. Slides were fixed in acetone for 10 min at room temperature and incubated 10 min with NH_4_Cl to reduce autofluorescence; nuclei were stained using 1 μg/ml DAPI (268298, Merck). Prolong Gold Antifade Reagent (Invitrogen) was used to mount the slides that were kept at 4°C until observation. Stained slides were observed with an LSM710 confocal laser scanning microscope, coupled to an AxioimagerM2 microscope (Zeiss). Micrographs were processed using ZEN (Zeiss) or Fiji software [23]. In both cases, inflammatory infiltration was estimated in binary images using the Fiji plugin for particle analysis and quantification.

### Electrocardiograph analysis

Mice were previously anesthetized with a single IP bolus of 25 mg/kg pentobarbital and 25 mg/kg ketamine. Electrocardiography (ECG) was performed using a bipolar system in which the electrodes were placed subcutaneously at the xiphoid cartilage (positive electrode), right shoulder (negative), and left shoulder as previously described [24]. Electrodes were connected to a Bioamp amplifier (AD Instruments, Bella Vista, Australia) and were digitalized through a PowerLab 8sp A/D converter (AD Instruments). Digital recordings were analyzed with Chart software for Windows v7.3.1 (AD Instruments), with events registered to 1 K/s and filtered to 60 Hz. Continuous ECG recordings were obtained for determining basal heart rate, defined as the point where there was no variation above 5%. At that point, 5mg/kg nitroglycerin (NG) was administered via IP to a group of mice, and subsequently 1.1 mg/kg isoproterenol, a non-selective beta adrenergic with positive chronotropic effects, was added IP to control mice and mice supplemented with fresh L-arginine mono-hydrochloride (3.75 mg/ml; Sigma-Aldrich) drinking water every other day. The register was followed until the end of the Iso effect evidenced by a decrease of the heart rate. Variation of heart rate and T/S waves with respect to pre-isoproterenol values were determined, and wave morphology was recorded. R and T axis measurements were based on the fact that cardiac depolarization and repolarization spreading constitute a vectorial magnitude. Depending on electrode placement, it is reflected in the ECG as positive or negative deflection or waves; R wave represents the principal vector of left ventricle depolarization and T wave is the electrical reflection of cardiac endocardial to epicardial repolarization vector. Based on that, it is possible to estimate the mean vector between two perpendicular bipolar and unipolar ECG leads (I vs aVF; II vs aVL; III vs aVR), graphing the R and T electrical values and determining the angle relative to the cardiac electrical center. Together, these measurements allow estimation of heart orientation.

### Statistical analysis

For *in vivo* experiments, data are shown as means ± SEM. Significance was evaluated by Student’s t-test when two groups were compared, by One-way ANOVA followed by the Tukey post-test for the analysis of parasitemia, and the Long-Rank (Mantel-Cox) test for survival using GraphPad Prism 5.00 software (La Jolla, CA, USA).

## Results

### *T*. *cruzi* infection depletes L-arginine and increases ADMA levels in plasma and heart tissue

**Fig 1:**
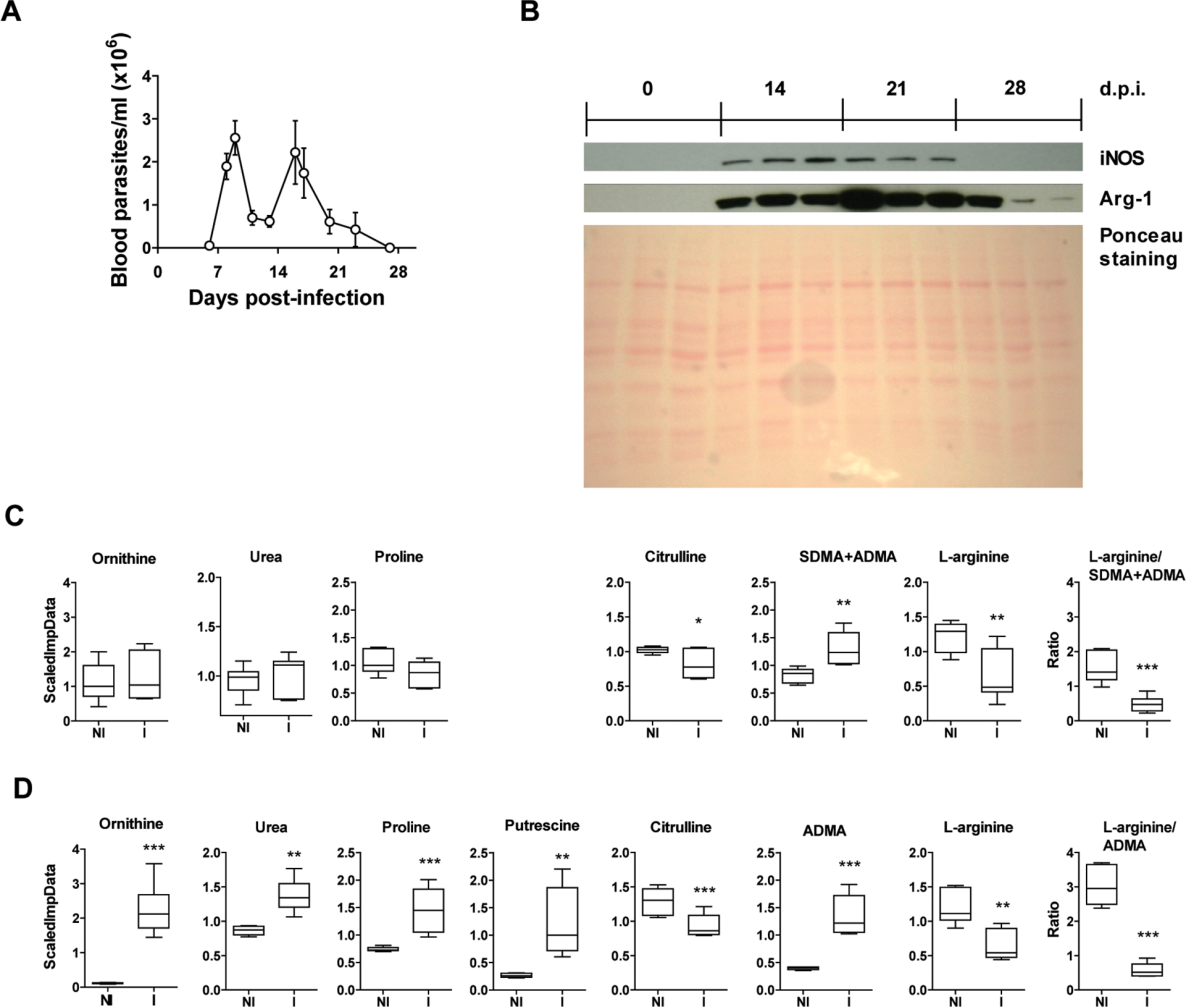
Analysis of L-arginine-derived metabolites in plasma and heart tissue during *T*. *cruzi* infection. Plasma and heart tissue were collected from BALB/c mice not infected (NI) or infected with a lethal dose of *T*. *cruzi* (I) and sacrificed at 21 d.p.i. **(A)** Parasitemia, as determined by direct counting under the optical microscope. **(B)** Western blot analysis of heart tissue extracts using antibodies against iNOS and Arg-1; Ponceau staining of the membrane is shown as a protein loading control. **(C)** Ornithine, urea, proline, citrulline, SDMA+ADMA, and L-arginine levels were determined in plasma and relative levels expressed as ScaledImpData as described in the Materials and Methods; the L-arginine/SDMA+ADMA ratio is also shown. **(D)** Ornithine, urea, proline, citrulline, ADMA, putrescine, and L-arginine levels were determined in heart tissue extracts; the L-arginine/ADMA ratio is also shown. Results from a single experiment are shown in box and whisker graphs; statistical analysis was performed using Student’s t-test (n = 6 mice per experimental group; *p<0.05; **p<0.005; ***p<0.001).

Infection of BALB/c mice with a lethal dose (2,000/mouse) of *T*. *cruzi* produced high levels of parasitemia (Fig 1A) and increased expression of iNOS and Arg-1 in heart tissue (Fig 1B), consistent with our previous reports [6, 18]. Urea cycle metabolites such as proline, ornithine, and urea are shown to illustrate collagen and polyamine synthesis during *T*. *cruzi* infection, and citrulline as an indirect measure of iNOS activity. At 21 d.p.i. there was a significant decrease in citrulline levels in the plasma and a significant increase in ADMA (Fig 1C), which is known to inhibit iNOS activity [25]. In addition, there was significant plasma L-arginine depletion and a decrease in the L-arginine/ADMA ratio (Fig 1C). Plasma levels of ornithine, urea and proline did not significantly change during infection.

Similarly, a significant decrease and increase in citrulline and ADMA, respectively, were seen in heart tissue at 21 d.p.i. (Fig 1D). However, levels of ornithine, urea, proline and putrescine levels also increased (Fig 1D). L-arginine and the L-arginine/ADMA ratio were also decreased in heart tissue upon infection (Fig 1D).

### L-arginine supplementation is beneficial for *T*. *cruzi* infected cells and mice

**Fig 2:**
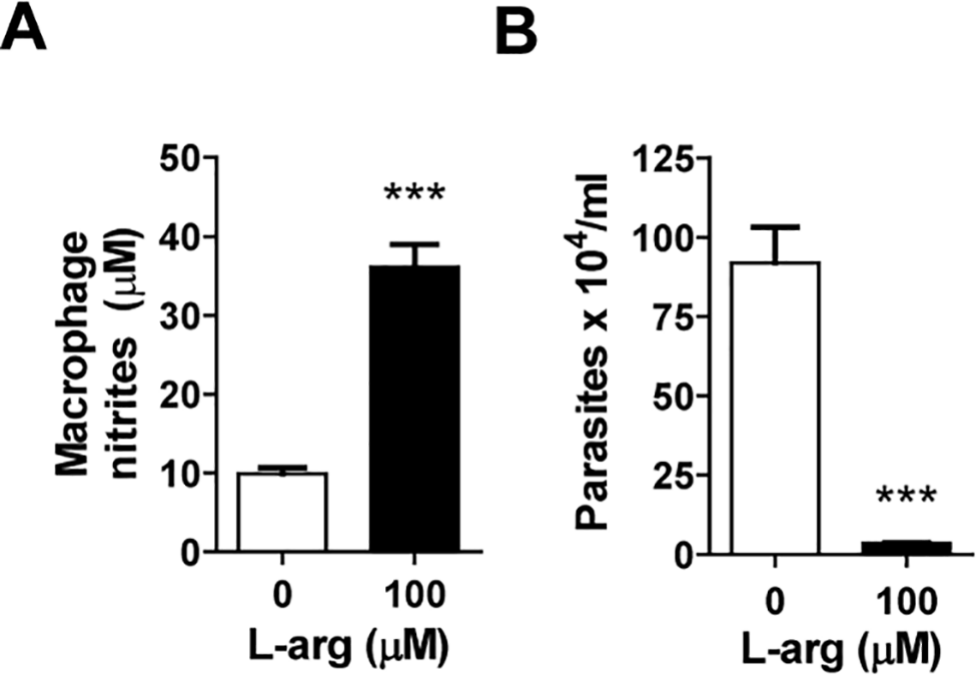
Effect of L-arginine on parasite load in infected macrophages *in vitro*. RAW 264.7 macrophages were infected with *T*. *cruzi* at a ratio 10:1, as described in the Materials and Methods. **(A)** Nitrite concentrations were determined by the Griess assay in culture supernatants at 72 h post-infection. **(B)** Parasite numbers in the culture medium were determined after 7 days post-infection. Mean ± SEM are shown; statistical analysis was performed using the Student’s t-test (***p<0.001).

To investigate the possible effects of reduced L-arginine levels on parasite replication, RAW 264.7 macrophages were infected with *T*. *cruzi*, and NO and parasite load were measured with or without L-arginine supplementation in the growth medium. The results showed that extracellular L-arginine is required for both NO production (Fig 2A) and intracellular parasite killing (Fig 2B).

**Fig 3:**
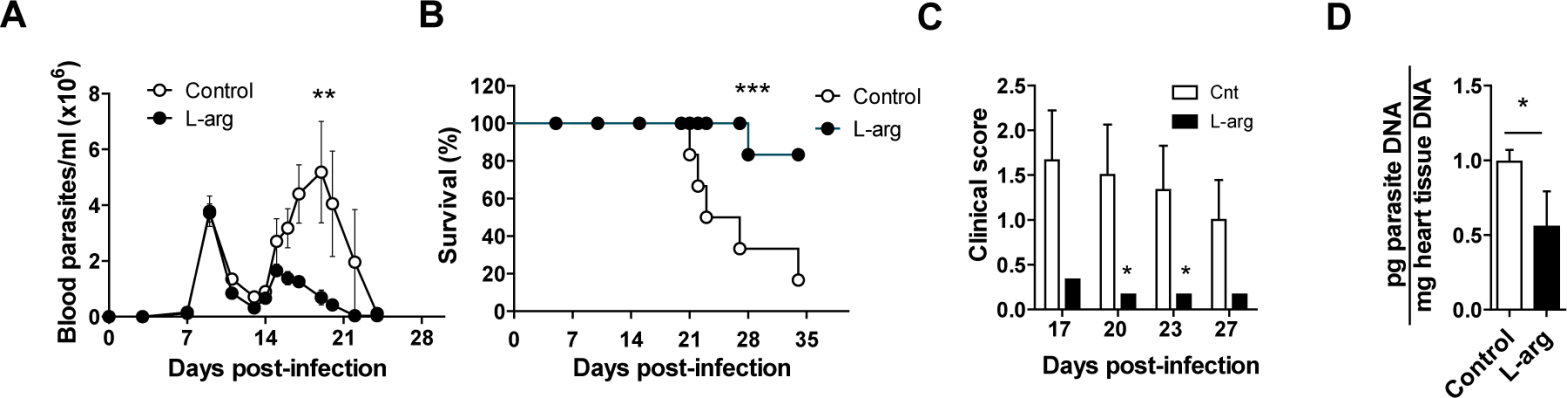
Effect of L-arginine supplementation on parasitological parameters of mice infected with *T*. *cruzi*. BALB/c mice were infected with *T*. *cruzi* and treated with L-arginine in the drinking water. Parasitemia, survival, clinical score and parasite load were monitored as described in the Materials and Methods. **(A)** Parasitemia was determined by the Brener method and analyzed by 1-way ANOVA. **(B)** Percent of survival was analyzed statistically using the Long-rank (Mantel-Cox) test. **(C)** Clinical score over the acute phase of infection. **(D)** Parasite load at 21 d.p.i. Data in C and D were analyzed using the Student’s t-test; mean ± SEM are shown from a representative experiment of at least 3 independent experiments (n = 6 mice per experimental group; *p<0.05; **p<0.005; ***p<0.001).

The above results lead us to investigate the effects of dietary L-arginine supplementation during *T*. *cruzi* infection in mice. For this, drinking water was supplemented with L-arginine, and parasitemia, survival, and clinical score were determined periodically through the end of the study at 21 d.p.i. L-arginine supplementation in infected mice caused a significant decrease in parasitemia (Fig 3A), and more strikingly a significant increase in survival (Fig 3B). In accordance with that, clinical scores during the peak of parasitemia (from 17–27 d.p.i.) were significantly lower in L-arginine-fed animals (Fig 3C), with a decrease in parasite load in the heart at 21 d.p.i. (Fig 3D).

In addition, L-arginine supplementation increased heart inflammation and decreased parasite burden, suggesting an enhanced immune response (S1 Fig).

**Fig 4:**
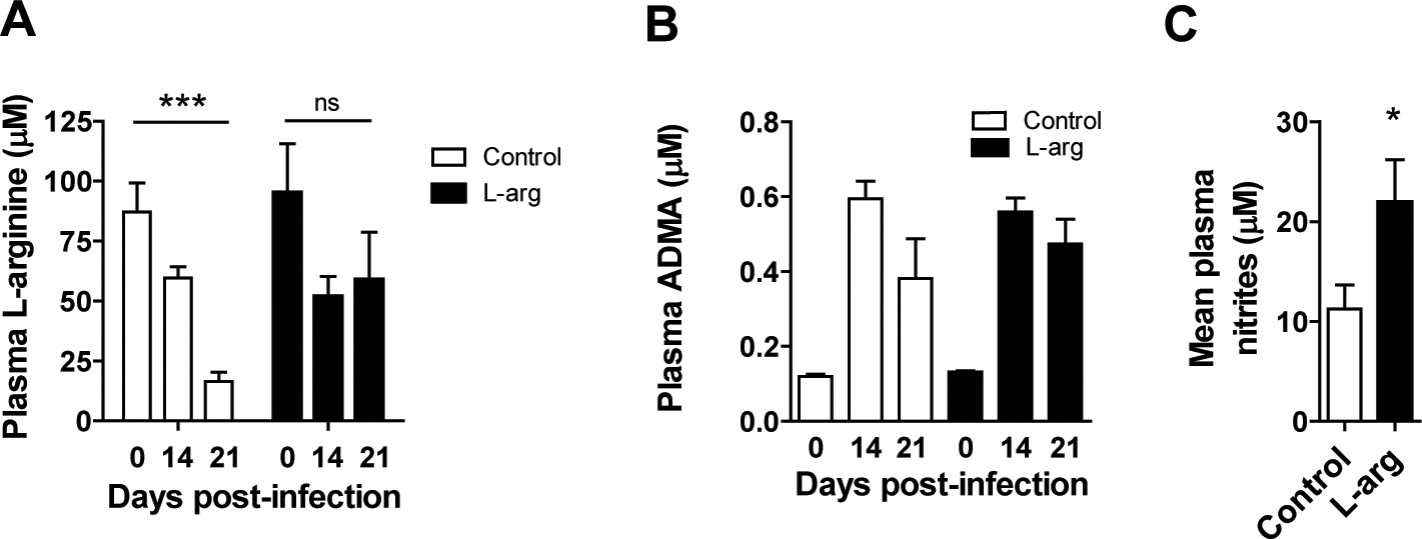
Effect of L-arginine supplementation on parasitological parameters and plasma urea cycle metabolites of mice infected with *T*. *cruzi*. BALB/c mice were infected with *T*. *cruzi* and treated (or not) with L-arginine in the drinking water. L-arginine, ADMA and nitrites were quantified in plasma as described in the Materials and Methods. **(A)** Plasma L-arginine concentration at different d.p.i. **(B)** Plasma ADMA concentration at different d.p.i. **(C)** Plasma nitrite concentration (mean of 0, 14 and 21 d.p.i.). Mean ± SEM are shown from a representative experiment out of at least 3 independent experiments; n = 6 mice per experimental group, except for (C) where 3 mice were analyzed at each time point; *p<0.05; **p<0.005; ***p<0.001.

Moreover, in infected mice L-arginine supplementation restored 61.8% of basal plasma L-arginine levels compared to 18.9% in non-treated controls at 21 d.p.i. (Fig 4A), without affecting the infection-induced increase in plasma ADMA levels (Fig 4B). Furthermore, plasma nitrites (mean of 0, 14 and 21 d.p.i.) were significantly higher in mice supplemented with L-arginine compared to non-treated controls (Fig 4C).

### Effect of pharmacological inhibition of iNOS during *T*. *cruzi* infection *in vivo*

**Fig 5:**
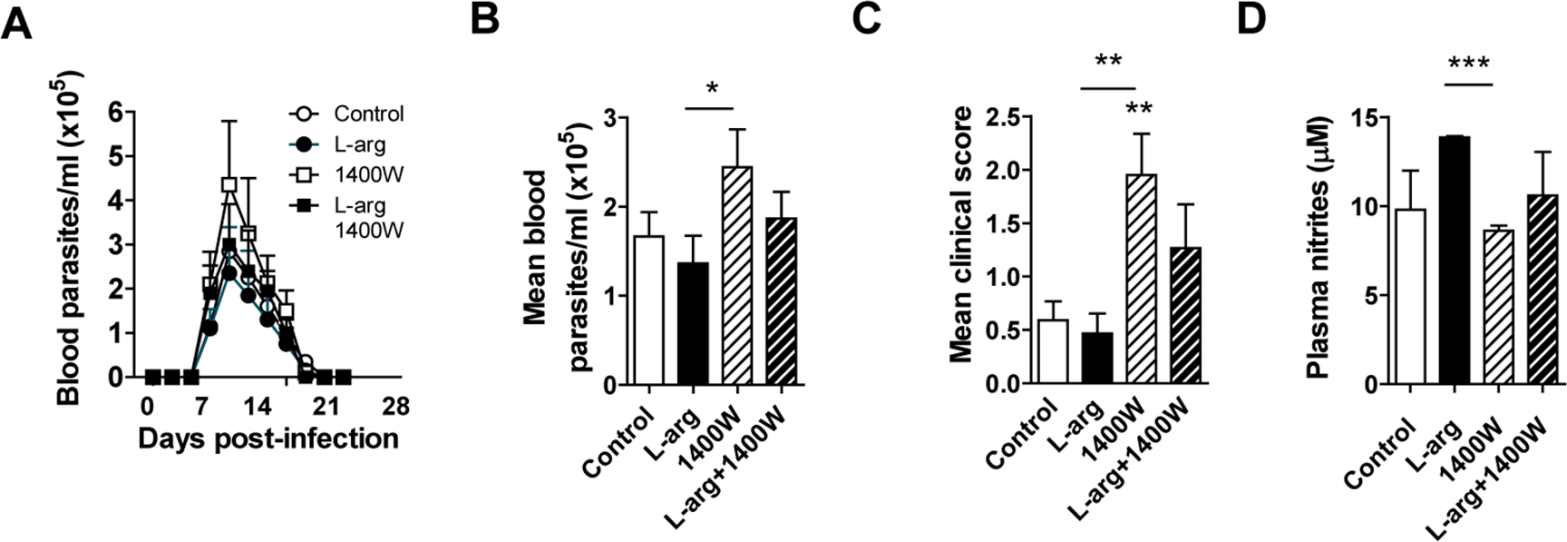
Effect of specific iNOS inhibitor 1400W in mice infected with *T*. *cruzi* with or without L-arginine supplementation. BALB/c mice were infected with *T*. *cruzi* and supplemented with L-arginine or not in the drinking water, and treated or not with 1400W by IP inoculation. Parasitemia, survival and clinical score were monitored and nitrites were determined in plasma by the Griess method. **(A)** Parasitemia was determined by the Brener method. **(B)** Mean parasitemia of 9, 11, 13 15, 17 and 19 d.p.i. **(C)** Mean clinical scores of 18, 19 and 21 d.p.i. **(D)** Plasma nitrite concentration at 21 d.p.i. Mean ± SEM are shown from an experiment (n = 6 mice per experimental group, except for (D) where 3 mice per time point were analyzed); *p<0.05; **p<0.005; ***p<0.001).

To investigate whether L-arginine supplementation had any effect on iNOS activity, we infected mice and treated them with L-arginine, alone or in combination with 1400W, a specific inhibitor of iNOS. This experiment was performed with a sublethal inoculum (50 parasites/mouse) to allow analysis of the beneficial and/or detrimental effects of the various compounds when combined. Despite the low initial dose, parasitemia reached a maximum of about 450,000 parasites per ml of blood (Fig 5A). Peak parasitemia (mean of 9, 11, 13, 15, 17 and 19 d.p.i.) and clinical scores (mean of 18, 19 and 21 d.p.i.) were higher in mice treated with 1400W than in other groups (Fig 5B and 5C, respectively). In addition, L-arginine supplementation was able to partially prevent the detrimental effect of iNOS inhibition by 1400W (Fig 5B and 5C). Finally, plasma nitrites were significantly higher at 21 d.p.i. in L-arginine-treated mice, an effect prevented by addition of 1400W (Fig 5D).

### L-arginine supplementation protected *T*. *cruzi*-infected mice from cardiac metabolic stress

**Fig 6:**
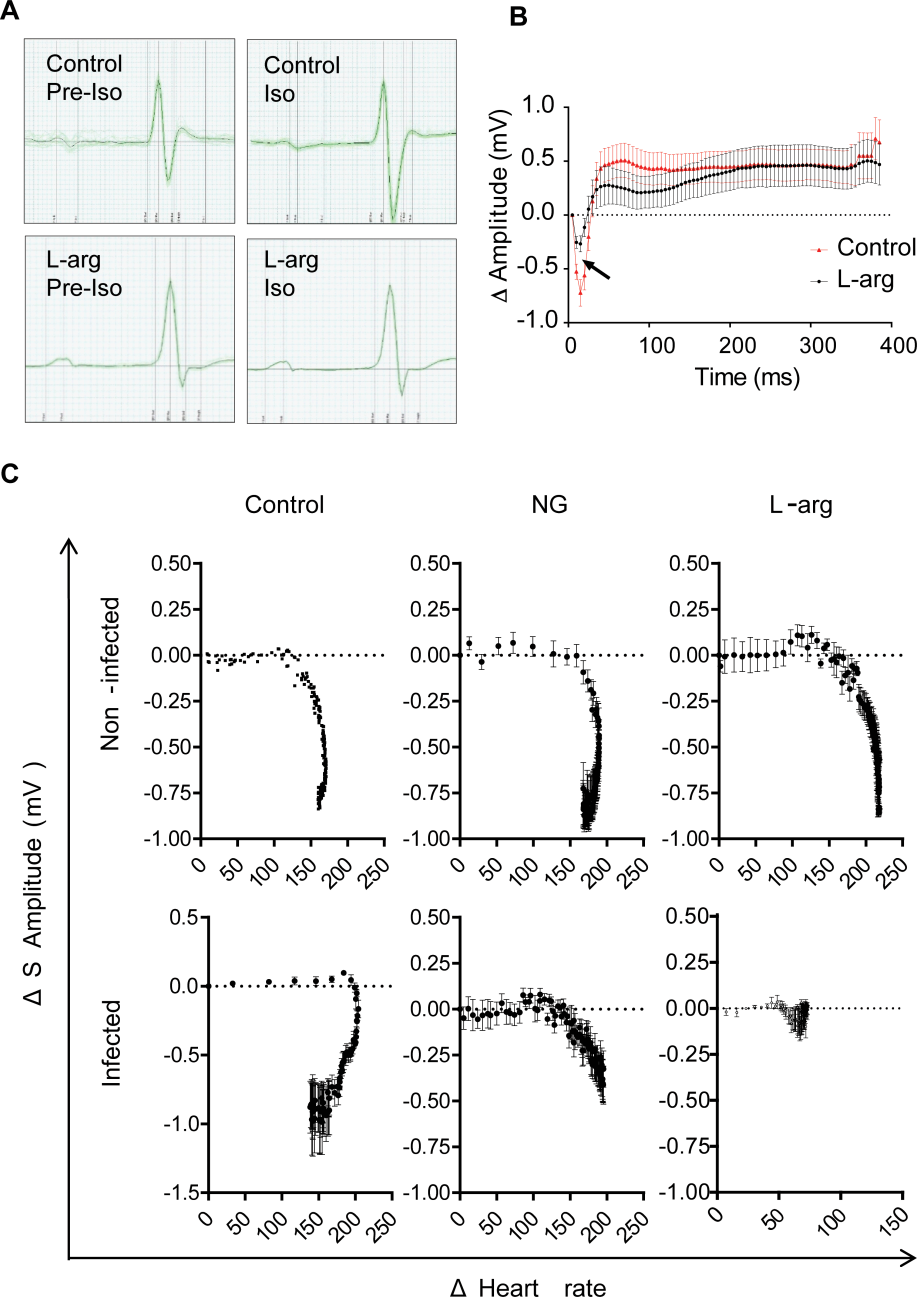
Effect of L-arginine supplementation on heart metabolic stress in *T*. *cruzi-*infected mice. BALB/c mice infected with *T*. *cruzi* were supplemented or not with L-arginine, and tested for heart performance under metabolic stress with 1.1 mg/kg of Isoproterenol (Iso) at 14 d.p.i. **(A)** Comparison of ECG traces of infected mice with or without L-arginine supplementation, both before (Pre-Iso) and after Iso treatment. **(B)** Graph showing mean ± SD of ΔS amplitude recorded after Iso treatment. **(C)**. ΔHeart rate was plotted against ΔS amplitude in non-infected (top) and infected mice (bottom). Additionally, treatment with NG (middle panels) and L-arginine (left panels) was performed; mean ± SEM are shown from an experiment performed with 4 mice per experimental group.

As cardiac disturbances are a hallmark of Chagas disease [1], the effects of L-arginine on cardiac function under metabolic stress conditions were analyzed. At 14 d.p.i., ECGs of infected mice were measured before (Pre-Iso) and after administration of isoproterenol (Iso), which increases the cardiac rate by binding to β1 adrenergic receptors that induce metabolic stress by reduction of coronary flux [26]. In addition, immediately before to Iso administration, we supplemented with a single bolus of NG, an NO donor. ECG measurements showed that the amplitude of the S wave in infected mice after Iso treatment changed dramatically, but not in those continuously supplemented with L-arginine (Fig 6A and S1 Video). Fig 6B shows the plot of mean ΔS amplitude (Iso S wave amplitude minus pre-Iso wave amplitude) of an ST segment selected five minutes after Iso treatment in infected control and L-arginine-treated mice. Fig 6C shows plots of ΔHeart rate [beats per minute (bpm) in Iso minus bpm in Pre-Iso] *versus* ΔS amplitude in six experimental groups (Control, NG and L-arginine both in non-infected and infected mice). Greater ΔS amplitude variations were observed in infected mice compared to the non-infected ones (Fig 6C, left panels). Moreover, in infected mice, the decrease in ΔS amplitude caused by Iso administration was partially reversed by NG, though it increased ΔHeart rate (Fig 6C, middle panels), a symptom of hypotension [27]. In contrast, infected mice, but not non-infected ones, showed decreased ΔS amplitude in response to L-arginine supplementation, as well as decreased ΔHeart rate (Fig 6C, right panels).

**Fig 7:**
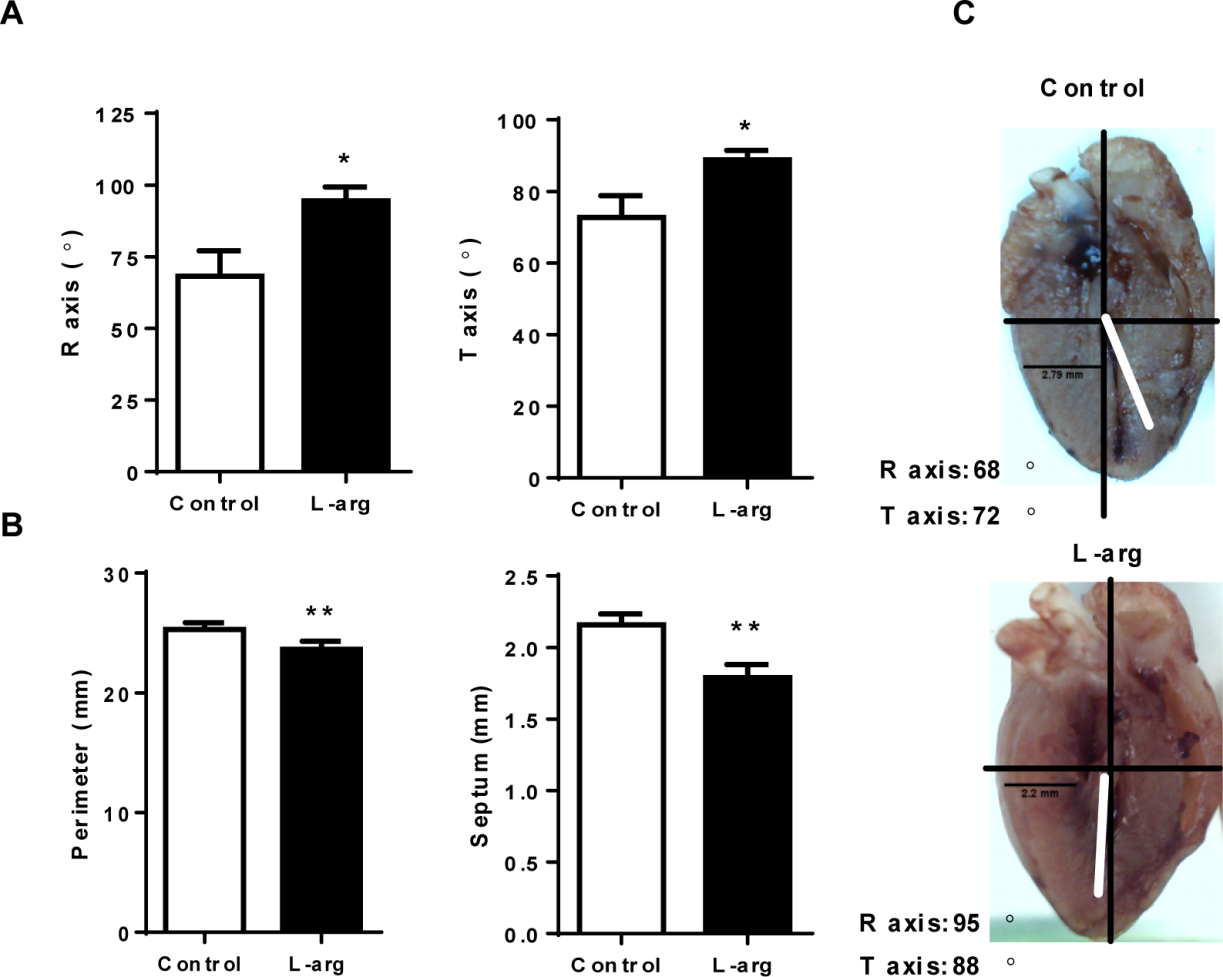
Effect of L-arginine supplementation on cardiac parameters of *T*. *cruzi-*infected mice. BALB/c mice were infected with *T*. *cruzi* and given drinking water with or without L-arginine supplementation. **(A)** The R and T axis were determined by ECG analysis using a bipolar system as described in the Materials and Methods; mean ± SEM are shown. **(B)** Heart perimeter and septum thickness measurements; mean ± SEM are shown. **(C)** Representative images of hearts of infected control (top) and L-arginine-supplemented (bottom) mice with a representation of the R and T axis from (A). The experiment was performed in 4 mice per group, and statistical analysis was done using the Student’s t-test (*p<0.05; **p<0.005; ***p<0.001).

Infection induced lateralization (changes in heart orientation) at 14 d.p.i., reflected by increased R and T axis angles (Fig 7A and 7C), which was prevented after L-arginine supplementation. In addition, heart perimeter and septum thickness were significantly decreased after L-arginine supplementation (Fig 7B and 7C).

## Discussion

Many physiologic and pathophysiologic processes are modulated by arginine availability [17]. We previously found elevated levels of Arg-1 in acute *T*. *cruzi* infection, associated with plasma L-arginine depletion [18]. Thus, it was hypothesized that L-arginine supplementation could be beneficial for infected mice. The results shown here indicate that there was an improvement in the disease outcome, evidenced by decreased parasite burden, higher survival, lower clinical scores and improved cardiac performance. Moreover, they suggest that L-arginine supplementation may be useful, either alone or in combination with other drugs for antiparasitic therapy. The observed beneficial effects of L-arginine supplementation may explain previous reports that it prevented vertical transmission in a rat model [28]. Although our study was done with a unique strain of the parasite, it is highly virulent and with cardiac tropism, characteristics that give further strength to our results. In addition, we very recently described the existence of common pathophysiologic patterns linked to clinical outcome of Chagas disease, conserved among the genetically diverse infecting strains, which suggests that our approach could be valid [29].

Levels of iNOS expression have been associated with parasite control, since NO is toxic for parasites, both extracellular and inside macrophages [11]. However, data from iNOS-deficient mice is controversial in the context of *T*. *cruzi* infection [30, 31]. Increased levels of iNOS expression were observed in the heart tissue of infected mice compared to non-infected mice, which correlates with a slight increase of NO in the plasma. Our results showed L-arginine depletion and high levels of ADMA after *T*. *cruzi* infection, pointing to them as potential biomarkers of pathology. In agreement, we found lower levels of citrulline (suggesting lower iNOS activity) during infection both in plasma and heart tissue. Lower L-arginine and higher ADMA may constrain iNOS activity, leading to insufficient NO production which is required for the control of parasite replication [11]. Thus, iNOS inhibition by metabolites such as ADMA could partially explain the conflicting results in the field, since iNOS expression might not always directly correlate with NO production, especially in highly virulent infections such as in this study.

Moreover, the heart levels of L-arginine were decreased and associated with an increase in ornithine, proline, putrescine and urea, likely indicating an activation of the polyamine pathway that may lead to pathological fibrosis and cardiac remodeling [32]. Altogether, our results suggest that NO levels in infected control mice did not increase enough to control parasite replication. Moreover, the L-arginine/ADMA ratio was greatly reduced during infection, likely contributing to pathology. Interestingly, a low L-arginine/ADMA ratio is also an indicator of vascular and cardiac alteration, and has been described as a predictor of NO bioavailability and mortality in dilated cardiomyopathy [33], a disease with some similarities with Chagasic cardiomyopathy.

One possible mechanism by which infection modulates intracellular and plasma ADMA levels may be increased protein degradation by parasite proteases. This would increase intracellular ADMA levels that could reach the extracellular milieu through cationic amino acid transporters (CATs), which are differentially expressed in tissues. CATs can also allow entry of ADMA into distant cells and tissues (reviewed in [34]). In *T*. *cru*zi infection, we have reported increased expression of CAT-1 and CAT-2b in heart tissue, likely expressed by infiltrating cells, in particular MDSCs [18]. CAT-1 and CAT-2b should allow cellular entry and exit of L-arginine and ADMA. In the endothelium, vasodilation can be stimulated by arginine (arginine paradox), which may be explained if eNOS (endothelial nitric oxide synthase isoform) activity is inhibited by ADMA, and relieved when the L-arg/ADMA ratio increases [35]. Thus, it is likely plasma ADMA rather than intracellular ADMA that determines NOS inhibition.

Macrophages are thought to be the most important effector cells in eliminating *T*. *cruzi* parasites, via a NO-mediated killing process [11]. Our results show that NO production in infected macrophages is strongly dependent on extracellular L-arginine, as described in other infections [36]. More interestingly, NO produced by enzymatic conversion of L-arginine by iNOS was needed for the elimination of parasites, indicating a crucial role of extracellular L-arginine for parasite killing, as previously described [12].

We also showed that dietary L-arginine supplementation significantly decreased parasitemia in infected mice, but more importantly reduced clinical scores by more than 80% while preventing death in 80% of the infected mice. This effect was associated with recovery of basal levels of L-arginine in the plasma compared to unsupplemented mice, and to an increase of NO production that in turn allows more efficient parasite killing.

Additionally, there was increased heart inflammation after L-arginine supplementation that likely contributed to lower parasite burden. This indicates a notable high beneficial effect of L-arginine supplementation in the outcome of the infection.

Since extracellular L-arginine levels clearly impacted iNOS-derived NO, we treated mice with the specific iNOS inhibitor 1400W, finding that iNOS inhibition significantly increased parasitemia and clinical score with respect to L-arginine supplemented mice. Despite this, there were no significant changes in NO production after 1400W treatment, though the combination of L-arginine slightly increased NO production compared to treatment with 1400W alone. This suggests NO production contributes to the beneficial effect mediated by L-arginine supplementation during acute *T*. *cruzi* infection.

Cardiac disturbances are a hallmark of Chagas disease [1], and the severe alterations of heart function evidenced by ECG records that are associated with the risk of sudden death [37] are very frequent in Chagas disease. ECG findings suggest that L-arginine supplementation also exerts a cardioprotective effect during *T*. *cruzi* infection. It has been described that pre-treatment with L-arginine can attenuate cardiac hypertrophy through regulation of key enzymes of the polyamine and NO production pathways [38], which were found to be altered in *T*. *cruzi* infection. Of note, L-arginine treatment has also been shown to improve isoproterenol-impaired basal left ventricular systolic function, likely mediated by NO production [39]. Also, morphological heart parameters significantly normalized after L-arginine supplementation in infected mice, leading to prevention of heart lateralization. There was also lower ΔS amplitude in infected compared to non-infected mice, and was dramatically decreased in mice infected and supplemented with L-arginine, suggesting an improvement in heart perfusion.

*T*. *cruzi* may well be affected by L-arginine supplementation, though unlike other kinetoplastids it is unable to utilize L-arginine for proliferation, and is insensitive to ornithine decarboxylase (ODC) inhibitors such as DFMO [40, 41] because it lacks ODC [42] and therefore cannot synthesize putrescine for proliferation. Instead, *T*. *cruzi* is dependent on polyamine uptake for growth and survival [42, 43]; thus, it stands to reason that L-arginine supplementation could enhance polyamine synthesis, which could be taken up by the parasite, increasing its proliferation. However, this is not the case as there is inhibition of parasite burden, indicating that L-arginine supplementation is primarily used by iNOS for NO production.

In summary, our results suggest that decreased levels of L-arginine and the presence of ADMA in plasma and tissues of infected hosts may be indicative of the severity of acute *T*. *cruzi* infection, and therefore are putative candidates for biomarkers of pathology. More importantly, our findings suggest that dietary supplementation with L-arginine in infected hosts, either alone or in combination with other antiparasitic drugs, may be useful for fighting infection, partially overcoming iNOS inhibition and allowing more efficient parasite killing by NO, while improving cardiac output, leading to increased survival and better clinical outcomes.

## Supporting information

S1 Video ECG records before and after isoproterenol administration. BALB/c mice were infected with *T*. *cruzi*, and tested for heart performance under metabolic stress with 1.1 mg/kg of Iso. ECGs were recorded at 14 d.p.i.; videos of Pre-Iso and Iso ECGs of control and L-arginine supplemented mice. **(A)** Control mouse. (B) Mouse with continuous supplement of L-arginine. ECGs from a representative mouse out of 4 for each group are shown.
(PPTX)

S1 Fig Effect of L-arginine supplementation on heart inflammation. Heart sections from BALB/c mice infected with the Y strain of *T*. *cruzi*, supplemented (L-arg) or not (Control) with L-arginine. **(A)** Qualitative H&E staining. **(B)** Inflammation was quantified in heart sections stained with DAPI using Fiji software [23]. Results from a representative mouse out of 4 for each group are shown.
(TIF)
