# Supplementary figures and images for: L-arginine supplementation reduces mortality and improves disease outcome in mice infected with Trypanosoma cruzi
Source: PLoS Negl Trop Dis. 2018 Jan 16;12(1):e0006179. doi: 10.1371/journal.pntd.0006179 (PMC5786330; doi:10.1371/journal.pntd.0006179)

## Slide 1
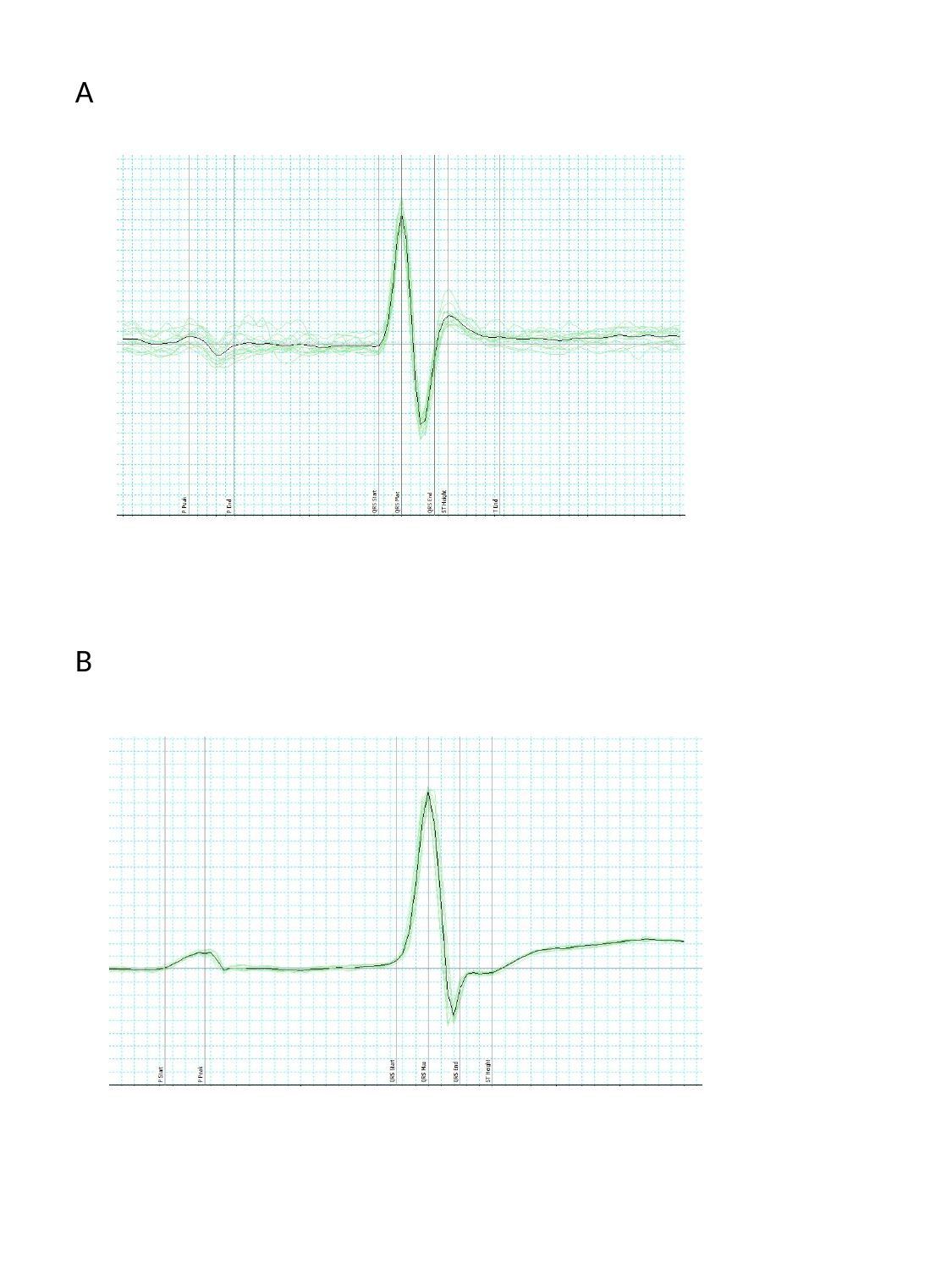

A
B

Supplement: S1 Video — BALB/c mice were infected with T. cruzi, and tested for heart performance under metabolic stress with 1.1 mg/kg of Iso. ECGs were recorded at 14 d.p.i.; videos of Pre-Iso and Iso ECGs of control and L-arginine supplemented mice. (A) Control mouse. (B) Mouse with continuous supplement of L-arginine. ECGs from a representative mouse out of 4 for each group are shown. (PPTX) [file pntd.0006179.s001.pptx]

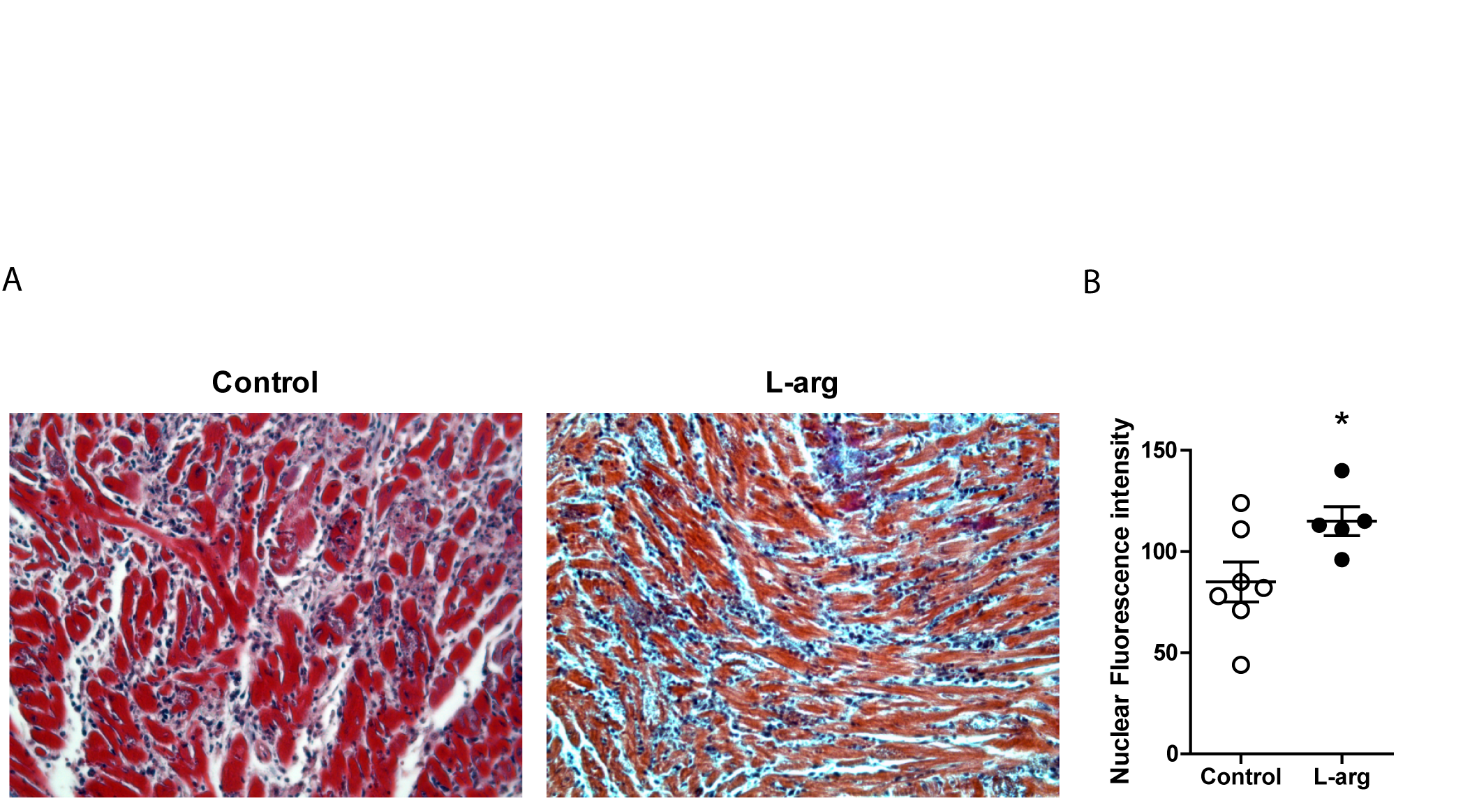

Supplement: S1 Fig — Heart sections from BALB/c mice infected with the Y strain of T. cruzi, supplemented (L-arg) or not (Control) with L-arginine. (A) Qualitative H&E staining. (B) Inflammation was quantified in heart sections stained with DAPI using Fiji software [23]. Results from a representative mouse out of 4 for each group are shown. (TIF) [file pntd.0006179.s002.tif]
